# Effectiveness and Safety of PD-1 Inhibitor Monotherapy for Elderly Patients with Advanced Non-Small Cell Lung Cancer: A Real-World Exploratory Study

**DOI:** 10.1155/2022/1710272

**Published:** 2022-07-22

**Authors:** Xiao-Ping Li, Wei-Dong Zhang, Ming-Jiang Li, Juan Wang, Jie Lian, Hong-Gang Zhou

**Affiliations:** ^1^Department of Thoracic Surgery, Tianjin First Central Hospital, School of Medicine, Nankai University, Tianjin, China; ^2^State Key Laboratory of Medicinal Chemical Biology, College of Pharmacy and Tianjin Key Laboratory of Molecular Drug Research, Nankai University, Haihe Education Park, Tianjin, China

## Abstract

**Background:**

Immunotherapy represented by PD-1 blockades had become the standard of care for advanced non-small cell lung cancer (NSCLC) gradually. Unfortunately, several PD-1 inhibitor-related studies excluded elderly patients with NSCLC over 75 years of age, resulting in relatively limited evidence regarding the efficacy and safety of PD-1 in elderly patients with NSCLC clinically.

**Objective:**

This study aimed to identify the effectiveness and safety of PD-1 blockade monotherapy among elderly patients with advanced NSCLC.

**Methods:**

Elderly patients with advanced NSCLC (≥65 years) who received PD-1 blockade monotherapy from September 2018 to December 2021 were screened retrospectively, and a total of 68 elderly patients with NSCLC were eligible for inclusion ultimately. The PD-1 blockades in the study were the available PD-1 monoclonal antibodies that had been approved for marketing in China, including camrelizumab, sintilimab, pembrolizumab, and nivolumab. The effectiveness and safety of the patients was collected retrospectively. Additionally, the correlation between prognosis and baseline characteristic subgroups was analyzed to identify the potential risk factors for progression-free survival (PFS).

**Results:**

The median age of the 68 elderly patients with advanced NSCLC was 73 years (range: 65–82 years). Best overall response during PD-1 blockade administration suggested that no patients were found with complete response, partial response was found in 14 patients, stable disease was noted in 29 patients, and 25 patients had progressive disease, yielding an objective response rate (ORR) of 20.6% (95%CI: 11.7%–32.1%) and a disease control rate (DCR) of 63.2% (95%CI: 50.7%–74.6%). Furthermore, prognostic analysis exhibited that the median progression-free survival (PFS) of the 68 patients with advanced NSCLC was 3.5 months (95%CI: 2.4–4.6) and the median overall survival (OS) was 10.5 months (95%CI: 6.3–14.7). Additionally, a total of 48 patients were observed with the treatment-related adverse reaction (70.6%) of the 68 elderly patients with NSCLC, and the incidence of grade 3 or above adverse reactions was 16.2%. Specifically, the most common adverse reactions were fatigue, diarrhea, rash, and abnormal liver function with the incidence of 25.0%, 22.1%, 16.2%, and 14.7%, respectively. Exploratory analysis between PFS and baseline characteristic subgroups suggested that ECOG performance status and number of metastatic lesions might be independent factors for PFS.

**Conclusion:**

PD-1 blockade monotherapy exhibited potential effectiveness and acceptable toxicity for elderly patients with NSCLC. ECOG performance status and number of metastatic lesions might be potential risk factors to predict the PFS of elderly patients with advanced NSCLC.

## 1. Introduction

Lung cancer is reported to be one of the most common solid tumors with almost 2.1 million new cases and 1.8 million deaths in 2018 worldwide [1]. Similarly, epidemiological data in China exhibited that there are approximately 0.81 million new cases and 0.71 million deaths of lung cancer annually [2]. Non-small cell lung cancer (NSCLC) is the predominant category with the prevalence of around 85% in lung cancer, which indicates that there are approximately 0.69 million new cases of NSCLC in China annually [3]. However, considerable patients with NSCLC in China were diagnosed with locally advanced or metastatic disease [4]. Noteworthily, the pathogenic genes of NSCLC were discovered and substantially investigated recently, and the targeted drugs were developed consecutively, making NSCLC with positive driver gene mutation the most successful cancer in precision medicine [5]. Furthermore, immunotherapy represented by programmed cell death 1 (PD-1) blockades also achieved convincing and durable efficacy for advanced NSCLC with negative driver gene mutation that was associated with worse prognosis previously, which improved the 5-year survival rate of patients with advanced NSCLC from 5% to 20% [6]. However, the overall prognosis of patients with metastatic NSCLC remained dismal, and more efficacious treatments were needed to be explored to augment the outcomes clinically.

To our knowledge, the occurrence of NSCLC increased with age and previous work suggested that the onset age of the diagnosis of NSCLC in clinical practice was approximately 70 years old [7]. Additionally, regarding the epidemiological data in China, a previous real-world study demonstrated that the proportion of patients older than 65 years old was 35.1%, highlighting that the proportion of elderly cases (>65 years) among all NSCLC patients in China was over 35% [8]. Unfortunately, given that most clinical trials set strict age screening criteria (usually <75 years), the proportion of patients over the age of 75 who were able to participate in clinical trials was probably less than 10% [9], resulting in limited available evidence as clinical guidelines among elderly patients with NSCLC [10]. The reason why elderly patients were excluded from most of the clinical trials could manifest as the following: older age, worse Eastern Cooperative Oncology Group (ECOG) performance status, absence of social support, cognitive impairment, accompanied with more comorbidities, and reluctance to receive therapeutic regimens with higher toxicity [11]. All these factors might contribute to the objective reasons regarding the restriction of elderly patients who participated in clinical trials clinically.

Additionally, PD-1 blockade monotherapy or in combination with chemotherapy had become the standard of care as second-line or first-line treatment for patients with advanced NSCLC, respectively [12]. PD-1 blockade monotherapy yielded an objective response rate (ORR) of approximately 20%, a median progression-free survival (PFS) of 3–6 months, and a median overall survival (OS) of 10–15 months as subsequent-line therapy among patients with previously treated NSCLC [13]. However, it should be noted that most of the patients in these trials were young patients. The median age of 690 patients with advanced NSCLC who received pembrolizumab monotherapy in the Keynote-010 trial was 63 years [14]. Additionally, the median age of 292 patients with non-squamous NSCLC who were treated with nivolumab in the Checkmate-057 trial was 61 years [15]. Similarly, among patients with advanced squamous NSCLC, the median age of 135 patients who received nivolumab monotherapy was 62 years [16]. Collectively, the current evidence-based studies of PD-1 blockade monotherapy in patients with advanced NSCLC was mainly focused on relatively young patients, suggesting that the efficacy and safety of PD-1 inhibitors in elderly NSCLC patients over 65 years was still scanty. Additionally, the therapeutic dilemma of PD-1 blockade monotherapy in clinical practice currently was the disappointing ORR [17]. Especially when the combined positive score (CPS) expression of programmed cell death ligand 1 (PD-L1) was less than 50%, the ORR of PD-1 blockade single agent was <20% [18]. As a result, it was necessary to identify the association between baseline characteristics and clinical outcomes of patients with NSCLC who were treated with PD-1 inhibitors, aiming to elucidate the potential patients that might benefit from PD-1 blockade administration.

Therefore, this study was to explore the real-world evidence regarding the efficacy and safety profile of PD-1 blockades among elderly patients with advanced NSCLC retrospectively.

## 2. Materials and Methods

### 2.1. Design of the Present Study

It seemed that considerable elderly patients with advanced NSCLC were treated with anlotinib monotherapy in China currently. As a result, this study was implemented as a retrospective study. Those who received PD-1 inhibitor single-agent therapy from September 2018 to December 2021 in the Department of Thoracic Surgery of the Tianjin First Central Hospital of Nankai University were screened consecutively. Besides, necessary eligibility criteria were adopted to present the clinical outcomes of PD-1 blockades for elderly patients with advanced NSCLC appropriately. The main inclusion criteria were: (1) diagnosis of NSCLC with the pathological staging of IIIb or IV; (2) age ≥65 years (age criterion of World Health Organization for elderly patients); (3) patients' performance status of 0–2 score; (4) patients progressed the previous systemic therapy and received PD-1 inhibitor single-agent therapy clinically; and (5) at least one measurable target lesion. The primary exclusion criteria manifested as: (1) patients were concomitant with a history of autoimmune disease or those were administering with steroids or other immunosuppressive drugs; (2) accompanied with one more tumor or serious disease that might threaten the survival determined by the investigators; and (3) patients were absent of a large number of baseline characteristics, or the diagnosis and efficacy assessment data were not available. However, those who were lost to follow-up were suitable for analysis in this study.

**Figure 1:**
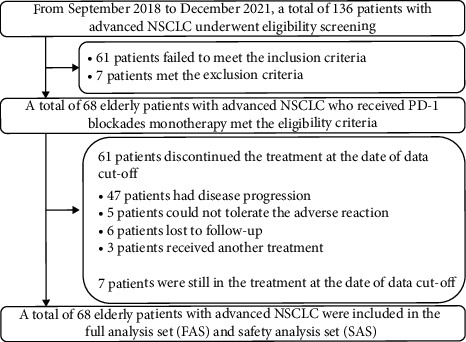
Study profile of this retrospective study.

The flow chart of the study is shown in Figure 1, and a total of 68 elderly patients with advanced NSCLC were included in the full analysis set (FAS) and safety analysis set (SAS) ultimately. Specifically, the efficacy endpoints included ORR, disease contrail rate (DCR), PFS, OS, and safety profile. Furthermore, this study was approved by the ethics committees of the Tianjin First Central Hospital of Nankai University. Written informed consent was obtained from all the patients included in this study in accordance with the recommendations of the Declaration of Helsinki.

### 2.2. Administration of PD-1 Blockades

Since this study was implemented as a retrospective study, all the elderly patients were treated with PD-1 inhibitor single-agent therapy in clinical practice. Therefore, PD-1 inhibitors were those approved in China mainland and available for Chinese patients in clinical practice, which consisted of camrelizumab (Jiangsu Hengrui Pharmaceutical Co. LTD), sintilimab (Innovent biopharmaceutical (Suzhou) Co., LTD), pembrolizumab (Merck (China) Co., LTD), and nivolumab (Bristol-myers Squibb (China) Investment Co. LTD). Camrelizumab, sintilimab, and pembrolizumab were intravenously administered with 200 mg on day 1, and nivolumab was intravenously administered with 360 mg on day 1; every three weeks was deemed as one therapeutic cycle. The treatment would be discontinued when disease progression or intolerable adverse reactions occurred.

### 2.3. Protocol of Assessment and Follow-Up

The iRECIST criteria were adopted to evaluate the therapeutic outcomes of the patients [19]. The available target lesions of the patients were evaluated using radiological methods such as CT or MRI scans at baseline and every two cycles when it was feasible. Complete response (CR) was termed as iCR, which was defined as the disappearance of all target and nontarget lesions, or nodal short-axis diameter <10 mm and no new lesions. Partial response (PR) was termed as iPR, which was defined as a decrease of ≥30% in tumor burden relative to baseline and non-unequivocal progression of nontarget lesions and no new lesions. Stable disease (SD) was termed as iSD, which was defined as neither PR nor PD. Progressive disease (PD) was classified as immune unconfirmed PD (iUPD) and immune confirmed PD (iCPD). IUPD was defined as an increase of ≥20% of the sum of longest diameters compared with nadir (minimum 5 mm) or progression of nontarget lesions or new lesion, and conﬁrmation of progression recommended minimum 4 weeks after the ﬁrst iUPD assessment. ICPD was defined as increased size of the target or nontarget lesions, increase in the sum of new target lesions >5 mm, progression of new nontarget lesions, or appearance of another new lesion [19].

Efficacy indicators were ORR and DCR, which were calculated based on the assessment of the best overall response during the treatment of PD-1 inhibitors. ORR was calculated by the proportion of CR and PR in FAS. DCR was calculated by the proportion of CR and PR and stable disease (SD) in FAS.

Additionally, baseline demographic characteristics and status of disease progression of each patient were collected. Follow-up was performed to obtain the prognostic data. The subsequent therapeutic regimens of the patients after the progression of PD-1 inhibitor administration were recorded, and the death status were mainly inquired through the communication with the patients' relatives, which was adopted from the previous study [20].

Additionally, Common Terminology Criteria for Adverse Events (CTCAE) version 5.0 criteria was adopted to evaluate the potential adverse reaction of the patients. And all the safety profile of the patients who were treated with PD-1 inhibitors was collected specifically to present the safety profile of PD-1 inhibitors among elderly patients with advanced NSCLC.

### 2.4. Statistical Analysis

All the data involved in the study were statistically carried out using SPSS (version 25.0). Statistical variables were presented as median (range) and number of patients (percentage) based on the corresponding data category. PFS and OS were defined according to the previous study [21]. Additionally, ORR and DCR and its 95% confidence interval (CI) were produced using the binomial exact method. Association between PFS and baseline characteristic subgroups was implemented using log-rank test, which yielded the median PFS value, 95% CI, and *P* value. Multivariate Cox regression analysis was adopted for PFS including the variables that were significant in univariate analysis. Those with no disease progression or death events at the date of data cutoff were treated as censored data. Frequency data were used in the safety analysis to estimate the incidence of the various adverse reactions. *P* < 0.05 was deemed as statistically significant.

## 3. Results

### 3.1. Baseline and Demographic Characteristics

Baseline and demographic characteristics of the 68 elderly patients with advanced NSCLC are illustrated in Table 1. All the patients included were elderly patients with the median age of 72 years (ranging from 65 to 82 years). Additionally, almost all the patients were negative of driven gene mutation, which included 38 patients with adenocarcinoma and 30 patients with squamous cell carcinoma. The other baseline and demographic characteristics are shown in Table 1, indicating that the 68 elderly patients included this study were the common elderly advanced NSCLC clinically.

### 3.2. Efficacy of the 68 Elderly Patients with Advanced NSCLC Who Received PD-1 Inhibitor Monotherapy

**Figure 2:**
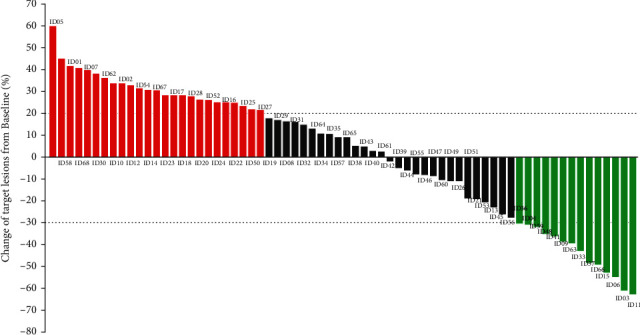
Waterfall plot regarding the change of target lesions from baseline among the 68 elderly patients with advanced NSCLC who received PD-1 inhibitor monotherapy individually (Green columns represent PR, black columns represent SD, and red column represents PD based on the best overall response).

**Figure 3:**
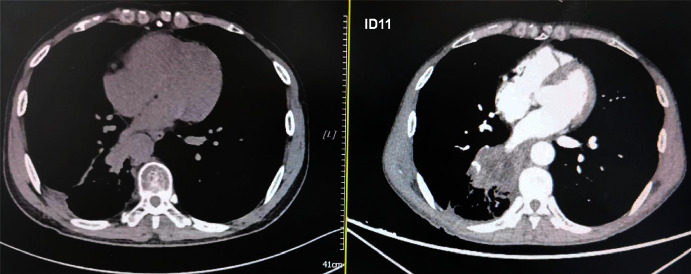
CT scan results of the changes for target lesions in the lung site of a female patient (PR) with advanced NSCLC before (a) and after the administration of sintilimab (b).

All the 68 elderly patients with NSCLC were available for the efficacy assessment. And the optimal response during PD-1 blockade administration was determined based on the efficacy assessment criteria of iRECIST. The efficacy outcome suggested that no patient had a CR, PR was observed in 14 patients, SD was noted in 29 patients, and PD was found in 25 patients, resulting in an ORR of 20.6% (95% CI: 11.7%–32.1%) and a disease control rate (DCR) of 63.2% (95%CI: 50.7%–74.6%). Specifically, the changes in the target lesions of the 68 patients after PD-1 inhibitor administration are exhibited in Figure 2. Obviously, the target lesions of some patients were shrunk dramatically and a total of 14 patients had achieved PR response after the treatment of PD-1 inhibitor monotherapy. Additionally, the comparison of the CT scans before and after sintilimab administration of one female patient is illustrated in Figure 3. The target lesions reduced significantly after the administration of sintilimab, which suggested that this patient benefited significantly from sintilimab administration.

### 3.3. Prognostic Outcomes of the 68 Elderly Patients with Advanced NSCLC Who Received PD-1 Inhibitor Monotherapy

**Figure 4:**
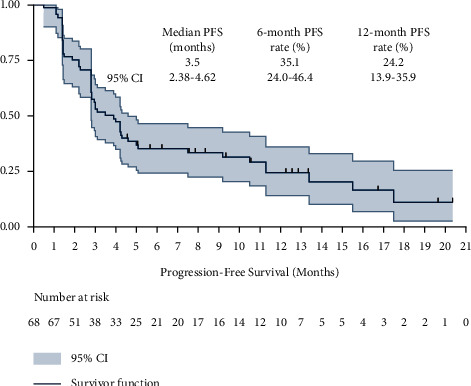
Progression-free survival of the 68 elderly patients with advanced NSCLC who received PD-1 blockade monotherapy.

Given that regular follow-up was performed for the patients in this study, the prognostic data of the majority patients were available. The data cutoff date of the present study was April 20, 2022, producing a median follow-up duration of the 68 elderly patients with advanced NSCLC of 9.8 months (follow-up range: 0.5–27.5 months). The PFS survival curve is exhibited in Figure 4, and the median PFS of the 68 elderly patients with advanced NSCLC who received PD-1 inhibitor monotherapy was 3.5 months (95%CI: 2.38–4.62). Furthermore, the 6-month PFS and 12-month PFS rate were 35.1% (95%CI: 24.0%–46.4%) and 24.2% (95%CI: 13.9%–35.9%), respectively. Noteworthily, a total of 10 elderly patients obtained a durable PFS benefit over 12 months.

**Figure 5:**
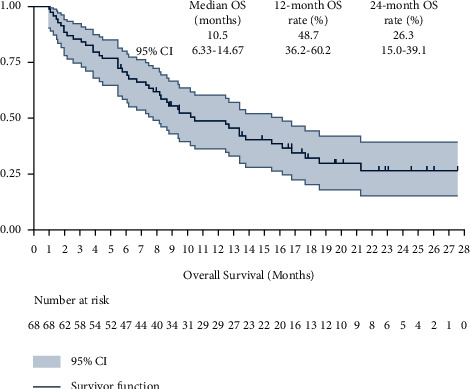
Overall survival of the 68 elderly patients with advanced NSCLC who received PD-1 blockade monotherapy.

Additionally, the relatively enough follow-up duration yielded an available OS data. Consequently, OS of the 68 elderly patients was also analyzed accordingly. The OS survival curve is shown in Figure 5, and the median OS of the 68 elderly patients with advanced NSCLC was 10.5 months (95%CI: 6.33–14.67). Additionally, the 12-month OS and 24-month OS rate were 48.7% (95%CI: 36.2%–60.2%) and 26.3% (95%CI: 15.0%–39.1%), respectively. Besides, a total of 10 elderly patients conferred a durable OS benefit over 20 months.

Furthermore, the correlation between PFS and baseline characteristic subgroups was analyzed, and the results presented by median PFS and 95% CI are illustrated in Table 2. Interestingly, ECOG performance status and number of metastatic lesions were significantly associated with PFS among the 68 elderly patients with advanced NSCLC, indicating that patients with ECOG performance status 0–1 score conferred a longer PFS than those with 2 score (median PFS: 4.2 vs 2.8 months, *P*=0.02), patients with number of metastatic lesions of ≤3 possessed a better PFS than those with number of metastatic lesions of >3 (median PFS: 3.9 vs 2.3 months, *P*=0.01). Those variables that showed statistically significant with the *P* value less than 0.05 in univariate analysis were included in the Cox multivariate analysis accordingly. As a result, ECOG performance status and number of metastatic lesions were included in the Cox multivariate analysis for adjustment of confounding factors, which is also shown in Table 2. Nevertheless, ECOG performance status score (HR = 0.69, *P*=0.03) and number of metastatic lesions (HR = 0.61, *P*=0.02) were also statistically significant after multivariate analysis, suggesting that ECOG performance status and number of metastatic lesions were independent factors for predicting the PFS of PD-1 inhibitor monotherapy among elderly patients with advanced NSCLC.

### 3.4. Safety Profile of the 68 Elderly Patients with Advanced NSCLC Who Received PD-1 Inhibitor Monotherapy

All the adverse reactions of the 68 elderly patients with advanced NSCLC during the PD-1 inhibitor monotherapy administration were collected as detailed as possible, and the results are illustrated in Table 3. A total of 48 patients were observed of different grade of adverse reactions (70.6%), and 11 patients' adverse reactions were deemed as grade ≥3 (16.2%). Of whom, one patient had dead from the PD-1 inhibitor-related pneumonitis after 2 months' administration of camrelizumab.

The common adverse reactions are shown in Table 3. Of which, adverse reactions with the grade ≥3 manifested as abnormal liver function (8.8%), fatigue (4.4%), diarrhea (2.9%), nausea and vomiting (2.9%), rash (1.5%), and pneumonitis (1.5%). Although one patient had experienced death of pneumonitis, the overall safety profile was tolerable and manageable.

## 4. Discussion

This study provided real-world evidence regarding the feasibility and tolerability regarding the administration of PD-1 inhibitor monotherapy for previously treated elderly patients with advanced NSCLC retrospectively. Simultaneously, the prognostic factors for PFS of PD-1 inhibitor monotherapy according to baseline characteristic subgroups were identified, and the results suggested that ECOG performance status and number of metastatic lesions were independent factors for PFS of PD-1 inhibitor monotherapy. Collectively, the findings of this study were of clinical guiding significance to provide the efficacy and safety data of PD-1 inhibitor monotherapy in clinical practice for elderly patients with advanced NSCLC.

In spite of the fact that the definition of elderly patients was controversial, elderly patients in our study were deemed as those over 65 years of age, which was in reference to the World Health Organization (WHO) criteria [22]. To our knowledge, the incidence of lung cancer in China is increasing year by year recently, and the estimated number of new cases each year was almost 815 thousand currently [23]. Among them, the elderly patients with lung cancer also increase dramatically. Unfortunately, the increase in age is concomitant with decreased body fat rate, decline in liver and kidney function, and other physical conditions [24]. All these factors based on age could compromise the absorption, metabolism, and excretion of PD-1/PD-L1 inhibitors [25]. As a result, elderly patients were usually excluded from most of the clinical trials, resulting in limited clinical evidence available for elderly patients with NSCLC in clinical practice [26]. Consequently, a large number of elderly patients with advanced NSCLC were in need of the efficacious and tolerable therapeutic options in clinical practice urgently to further prolong their survival [27].

Of the 68 elderly patients included in our study, a total of 13 patients were treated with PD-1 inhibitors in second-line therapy and the rest 55 patients received PD-1 blockades in third line or more lines. Given that some PD-1 blockades were licensed as second-line monotherapy for patients with advanced NSCLC in China, the administration of PD-1 inhibitors in our study was reasonable and ethical. The efficacy outcomes indicated that the ORR and DCR of the 68 elderly patients with advanced NSCLC who received PD-1 inhibitors were 20.6% and 63.2%, respectively. The median PFS was 3.5 months and the median OS was 10.5 months; it seemed that the ORR and PFS outcomes in our study were consistent with the ORR and PFS of Checkmate-017 and Checkmate-057 regarding nivolumab as the second-line treatment of squamous cell NSCLC and non-squamous NSCLC, respectively (ORR was approximately 20%, and median PFS was almost 3 months) [15, 16]. Besides, the second-line monotherapy with pembrolizumab in advanced NSCLC could also achieve an ORR of 18% and a median PFS of 3.9 months according to the Keynote-010 trial [14], which was in concert with that in our study to some extent. Interestingly, it should be noted that the median PFS in Keynote-010 was slightly longer than the median PFS in our study. The potential reasons might be attributed in two aspects: on the one hand, the discrepancy of age between the two studies might contribute to the survival difference. The median age in Keynote-010 and our study was 63 years and 72 years, respectively. The previous study had indicated that older age was usually correlated with worse prognosis to some extent [28]. On the other hand, it should be noticed that all the patients included in the Keynote-010 trial was the ECOG performance status of 0–1 score. However, ECOG performance status of score 2 in our study accounted for 30.9%. The previous relevant study had elucidated that ECOG performance status was an independent factor to involve in the PFS and OS for patients with advanced NSCLC [29]. And the results of the multivariate Cox analysis in our study suggested that patients with ECOG of score 2 conferred a worse prognosis. Furthermore, our study was designed as a retrospective analysis. Management of the patients in the retrospective study was not sufficient and normative compared with a well-designed clinical trial, thus deteriorating the efficacy and prognosis in our study to some extent, which was also proved by the previous retrospective study among patients with advanced NSCLC [30]. The above three aspects might be the potential reasons why the median PFS in our study is inferior to that in the Keynote-010 study. Additionally, we noticed that another retrospective study launched by Giulia *G* et al. investigated the efficacy and safety of PD-1 inhibitor in elderly patients among the Italian population that included 180 patients younger than 70 years and 110 patients older than 70 years [31]. The results indicated that patients with advanced NSCLC older than 70 years who received immunotherapy might achieve an ORR of 20%, a median PFS of 3.5 months, and a median OS of 11.3 months, which was in line with the clinical outcomes in our study. Another Italian, retrospective study initiated by Andrea luciani et al. recruited a total of 86 patients with advanced NSCLC aged ≥75 years who were administered with PD-1 inhibitors [32]. Clinical outcomes suggested that the PD-1 inhibitor regimen produced a DCR of 65.1%, a median PFS of 5.6 months, and a median OS of 10.1 months. It seemed that the median PFS was better than that in our study. This discrepancy might be attributed to the heterogeneity of patients included [33]. Collectively, all the above studies indicated that PD-1 inhibitor monotherapy might be an efficacious therapeutic option for elderly patients with advanced NSCLC in real-world practice.

Noteworthily, the exploratory analysis between PFS and baseline characteristic subgroups in our study indicated that elderly patients with advanced NSCLC might benefit from PD-1 inhibitor monotherapy uniformly regardless of the majority baseline characteristic subgroups, which highlighted that the effectiveness of PD-1 blockades was stable and balanced across different baseline characteristic subgroups [34]. Nevertheless, it seemed that ECOG performance status and number of metastatic lesions were independently associated with PFS in multivariate Cox analysis, which was consistent with the previous retrospective study [35]. Noteworthily, the previous relevant study had indicated that patients with ECOG performance status of score 2 and metastatic lesions of >3 trended to confer an inferior prognosis [11, 36]. Collectively, whether number of metastatic lesions and ECOG performance status might be used as prognostic biomarkers for elderly patients with advanced NSCLC who received the treatment of PD-1 blockade monotherapy should be validated in prospective clinical trials subsequently.

Furthermore, the safety profile of the 68 elderly patients with advanced NSCLC who received PD-1 inhibitor monotherapy was also presented in this study. And the results exhibited that the incidence of adverse reactions was 70.6% among the 68 elderly patients who received PD-1 monotherapy. Of whom, the incidence of adverse reaction with grade ≥3 was only 16.2%, which indicated that the regimen of PD-1 inhibitor administration was acceptable and manageable. And the safety profile in our study was consistent with the adverse reactions of the previous retrospective study regarding PD-1 blockades in patients with advanced NSCLC [8]. However, some immunotherapy-related adverse reactions should be paid more attention to the elderly patients who received PD-1 inhibitor monotherapy [37]. Firstly, we observed one patient dead from pneumonitis after 2 months' therapy of camrelizumab, which suggested that more active measures should be taken when the elderly patients received PD-1 inhibitors to attenuate the potential severe pneumonitis. Secondly, we also noticed that a total of 10 patients experienced abnormal liver function, of whom 6 patients were deemed as grade ≥3 ASL/ALT elevation, which was slightly higher than that observed in the Keynote-010 trial [14]. We speculated that the possible explanation might be the fact that elderly patients usually tended to have a relatively poor liver and kidney function own to more underlying comorbidities, thus potentiating the liver function abnormalities after the administration of PD-1 inhibitor monotherapy. As a result, the safety of elderly patients who received PD-1 blockade monotherapy was controllable. And more attention should be paid to the pneumonitis and liver function. And the safety data should also be validated in prospective clinical trials subsequently.

Obviously, some potential limitations existed in our study inevitably. Firstly, the primary limitation was that the sample size was relatively small as a real-world study—only 68 elderly patients were included for analysis. Clinical outcomes and adverse reactions of PD-1 blockade single agent were needed to be confirmed in more elderly patients with advanced NSCLC. Additionally, multi-PD-1 blockades were administered for elderly patients, which might result in heterogeneous and diverse efficacy. Besides, PD-L1 expression failed to be tested to select the potential elderly patients that might benefit from PD-1 administration.

## 5. Conclusion

This study provided real-world evidence regarding the feasibility and tolerability regarding the administration of PD-1 inhibitor monotherapy for previously treated elderly patients with advanced NSCLC retrospectively. And the prognostic factors for PFS of PD-1 inhibitor single agent in baseline characteristic subgroups suggested that ECOG performance status and number of metastatic lesions were independent factors for predicting the PFS of PD-1 inhibitor monotherapy. The conclusion should be elucidated in prospective trials subsequently.

## Figures and Tables

**Table 1:** Baseline and demographic characteristics of the 68 elderly patients with advanced NSCLC.

Baseline characteristics	Total (*N* = 68)	Percentage (%)
*Age (year)*		
Median (range)	72 (65–82)	

*Gender*		
Male	43	63.2
Female	25	36.8

*ECOG performance status*		
0–1	47	69.1
2	21	30.9

*Pathological staging*		
IIIb	8	11.8
IV	60	88.2

*Smoking status*		
Nonsmoker/former smoker	49	72.1
Smoker	19	27.9

*Histology*		
Adenocarcinoma	38	55.9
Squamous cell carcinoma	30	44.1

*Lines of PD-1 blockade therapy*		
Second line	13	19.1
Third line or more	55	80.9

*Number of metastatic lesions*		
≤3	45	66.2
>3	23	33.8

*History of targeted drug therapy*		
Yes	32	47.1
No	36	52.9

*PD-1 blockades*		
Camrelizumab	23	33.8
Sintilimab	20	29.4
Pembrolizumab	16	23.5
Nivolumab	9	13.2

NSCLC, non-small cell lung cancer; PFS, progression-free survival; ECOG, Eastern Cooperative Oncology Group; PD-1, programmed cell death protein 1.

**Table 2:** Association analysis between PFS of the 68 elderly patients with advanced NSCLC and baseline characteristic subgroups in univariate analysis and multivariate Cox analysis.

Baseline characteristics	Median PFS (95%CI)	*P* (univariate analysis)	Multivariate analysis	
			HR (95%CI)	*P*
*Age*				
<72	3.5 (2.43–4.57)	0.53		
≥72	3.1 (2.39–3.81)			

*Gender*				
Male	3.1 (2.39–3.81)	0.32		
Female	3.9 (2.61–5.19)			

*ECOG performance status score*				
0–1	4.2 (2.31–6.09)	0.02	0.69 (0.41–0.91)	0.03
2	2.8 (2.11–3.49)			

*Pathological staging*				
IIIb	3.9 (2.85–4.95)	0.44		
IV	3.1 (2.18–4.02)			

*Smoking status*				
Nonsmoker/former smoker	3.9 (2.91–4.89)	0.53		
Smoker	3.5 (2.62–4.38)			

*Histology*				
Adenocarcinoma	3.5 (2.41–4.59)	0.42		
Squamous cell carcinoma	3.1 (2.31–3.89)			

*Lines of PD-1 blockade therapy*				
Second line	3.9 (2.75–5.05)	0.67		
Third line or more	3.5 (2.48–4.52)			

*Number of metastatic lesions*				
≤3	3.9 (2.38–5.42)	0.01	0.61 (0.37–0.83)	0.02
>3	2.3 (1.42–3.18)			

*History of targeted drug therapy*				
Yes	3.9 (3.01–4.79)	0.42		
No	3.5 (2.46–4.54)			

*PD-1 blockades*				
Camrelizumab	3.1 (2.08–4.12)	0.36		
Sintilimab	3.5 (2.81–4.19)			
Pembrolizumab	3.9 (2.71–5.09)			
Nivolumab	3.0 (2.03–3.97)			

NSCLC, non-small cell lung cancer; PFS, progression-free survival; ECOG, Eastern Cooperative Oncology Group; PD-1, programmed cell death protein 1; CI, confidence interval; HR: hazard ratio.

**Table 3:** Safety profile of the 68 elderly patients with advanced NSCLC who received PD-1 inhibitor monotherapy.

Adverse reactions	Total (*N*, %)	Grade 1–2 (*N*, %)	Grade ≥3 (*N*, %)
Adverse reactions	48 (70.6)		11 (16.2)
Fatigue	17 (25.0)	14 (20.6)	3 (4.4)
Diarrhea	15 (22.1)	13 (19.2)	2 (2.9)
Rash	11 (16.2)	10 (14.7)	1 (1.5)
Abnormal liver function	10 (14.7)	4 (5.9)	6 (8.8)
Nausea and vomiting	7 (10.3)	5 (7.4)	2 (2.9)
Pneumonitis	5 (7.4)	4 (5.9)	1 (1.5)
RCCEP	5 (7.4)	5 (7.4)	0 (0.0)
Fever	3 (4.4)	3 (4.4)	0 (0.0)
Stomatitis	2 (2.9)	2 (2.9)	0 (0.0)

NSCLC, non-small cell lung cancer; RCCEP: Reactive cutaneous capillary endothelial proliferation; PD-1, programmed cell death protein 1.

## Data Availability

The data generated during this study can be obtained from the corresponding authors upon reasonable request.
